# Identification of *Lygus hesperus* by DNA Barcoding Reveals Insignificant Levels of Genetic Structure among Distant and Habitat Diverse Populations

**DOI:** 10.1371/journal.pone.0034528

**Published:** 2012-03-30

**Authors:** Changqing Zhou, Irfan Kandemir, Douglas B. Walsh, Frank G. Zalom, Laura Corley Lavine

**Affiliations:** 1 Department of Entomology, Washington State University, Pullman, Washington, United States of America; 2 Department of Biology, Ankara University, Ankara, Turkey; 3 Irrigated Agriculture Research and Extension Center, Prosser, Washington, United States of America; 4 Department of Entomology, University of California Davis, Davis, California, United States of America; Auburn University, United States of America

## Abstract

**Background:**

The western tarnished plant bug *Lygus hesperus* is an economically important pest that belongs to a complex of morphologically similar species that makes identification problematic. The present study provides evidence for the use of DNA barcodes from populations of *L. hesperus* from the western United States of America for accurate identification.

**Methodology/Principal Findings:**

This study reports DNA barcodes for 134 individuals of the western tarnished plant bug from alfalfa and strawberry agricultural fields in the western United States of America. Sequence divergence estimates of <3% reveal that morphologically variable individuals presumed to be *L. hesperus* were accurately identified. Paired estimates of F_st_ and subsequent estimates of gene flow show that geographically distinct populations of *L. hesperus* are genetically similar. Therefore, our results support and reinforce the relatively recent (<100 years) migration of the western tarnished plant bug into agricultural habitats across the western United States.

**Conclusions/Significance:**

This study reveals that despite wide host plant usage and phenotypically plastic morphological traits, the commonly recognized western tarnished plant bug belongs to a single species, *Lygus hesperus*. In addition, no significant genetic structure was found for the geographically diverse populations of western tarnished plant bug used in this study.

## Introduction

The *Lygus* bug complex in the United States and Canada is an economically important insect group in a diversity of cropping systems including cotton, vegetables, fruit, pulse crops and seed crops. In the United States, there are three recognized major *Lygus* species: *L. lineolaris*, *L. elisus* and *L. hesperus* among approximately 28 morphologically similar *Lygus* species [1]. *Lygus hesperus* Knight, also called the western tarnished plant bug, has a wide distribution and has been documented to use at least 25 crop plants and 117 non-crop plants as hosts [1], [2]. It is the key perennial direct pest in alfalfa produced for seed, strawberry, conifer nurseries, cotton, and pulse crops [1]. Alfalfa is one of the major host plants of Lygus species in terms of land area - the United States alfalfa seed crop alone is worth approximately $14 million annually, while the alfalfa forage crop is worth $267 million annually [3]. Most alfalfa seed production is in the western United States. In Washington state, 5,666 hectares of alfalfa seed and 180,490 hectares of alfalfa forage are planted annually [3]. It is the concentration of feeding on reproductive parts that makes *Lygus* species some of the most insidious pests of seed crops [4]. Strawberries are the fourth highest ranked U.S. fruit in terms of value of production, following grapes, oranges and apples, and California production in 2010 was valued at nearly $1.8 billion [5]. Lygus species damage strawberry fruit in the western US and Canada by feeding on individual achenes of the developing strawberry fruit resulting in the deformation known as ‘catfacing’ [6]. Lygus damage to fruit can exceed 30 percent if populations are not controlled [7].

In the western U.S.A., *L. hesperus* is the dominant species in a complex of closely related plant bugs that includes *L. elisus*, *L. shulli*, and *L. lineolaris* [1]. Insecticide resistance has been a continuing issue for Lygus control as populations of *L. lineolaris* in the southeastern U.S.A. and populations of *L. hesperus* in the western US have developed tolerance to organophosphate and pyrethroid insecticides [8], [9]. Increased environmental concerns, coupled with the growing prevalence of insecticide resistance and outbreaks of secondary pests have led to an increased interest in integrated pest management and biological control [10]. Improvements of IPM strategies for *L. hesperus* control necessitate better identification tools for *Lygus* species. Many species of *Lygus* are morphologically similar, taxonomic character differences are subtle, and in some species there is a wide range of ontogenetic and geographic variation [1]. The extent to which differences exist among these closely related species in their susceptibility to insecticides, host plant preference, intensity of parasitism by parasitoid wasps, and level of predation from a complex of generalist predatory beneficial insects within and among regional cropping systems needs to be identified for IPM and biocontrol strategies [1], [8]–[9]. Thus, one of the most important unsolved problems for the efficient control of *Lygus* bugs is accurate species identification.

Morphological and molecular characters are useful when used in combination to identify morphologically similar species. DNA barcoding is the term used to describe the use of a short standardized DNA region as a species identification tool [11]. DNA barcoding is based on the assumption that each species has a unique DNA barcode and the genetic variation between species is greater than that within the species [11]. A major goal of DNA barcoding is to aid the clarification of current taxonomic problems [12]–[15]. Currently, in most animals, a fragment of the mitochondrial cytochrome c oxidase I (CO1) gene is used as the standard barcoding region, including for true bugs [11]–[15].

The western tarnished plant bug has long been considered to be an extremely morphologically variable species. *Lygus hesperus* Knight has a wide distribution across western North America in a diversity of agricultural and low elevation regions extending from southern British Columbia to northern Mexico including northern Baja California [1]. It remains unknown if the differences in color within populations and across its distribution are simply a result of phenotypic plasticity or caused by genetic differentiation or even speciation. Its large-range, disjunct distribution, and occupation of a diversity of agricultural and non-agricultural habitats suggest that populations of *L. hesperus* may have quantifiable genetic differences. To investigate this problem, this study had three main objectives. The first was to examine DNA sequences from different *L. hesperus* populations across its range to compare *L. hesperus* to two other closely related species of *Lygus - Lygus elisus* and *Lygus lineolaris*. The second was to investigate the evidence for cryptic speciation within populations of *L. hesperus* across its range using DNA barcoding. Finally, we examined differences in *L. hesperus* populations from California, Washington, and Idaho to ascertain the likely level of movement and gene flow between populations. The results of these studies are presented here.

## Methods

### Insect Collection

**Figure 1:**
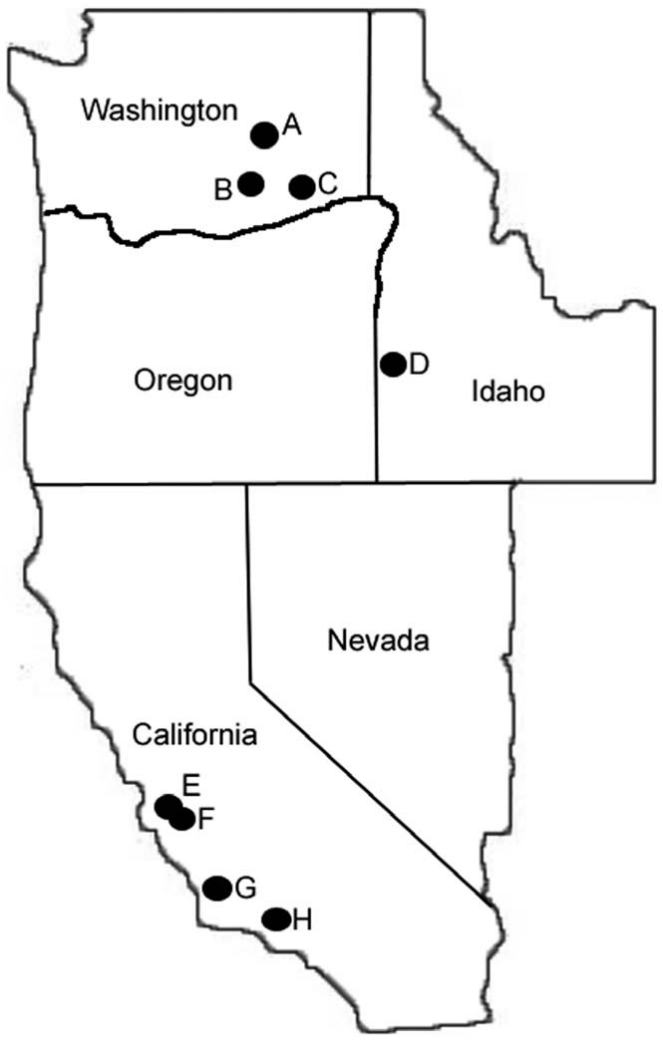
Map of collection sites in Washington, California, and Idaho. A: Othello, WA. B: Prosser, WA. C: Touchet, WA. D: Parma, ID. E: Watsonville, CA. F: Prunedale, CA. G: Santa Maria, CA. H: Camarillo, CA.

The western tarnished plant bug is a polyphagous insect on a variety of host plants. Identification of *Lygus hesperus* is typically done by field collection and morphological identification by growers, extension entomologists, and research entomologists in the field and in the laboratory. To validate morphological identification in the field with our genetic data, we collected hundreds of adult western tarnished plant bugs at different times of the summer growing season with a sweep net and by hand from alfalfa (Washington and Idaho) and strawberry (California) in 2009 and 2010 (Table 1; Figure 1). We chose these locations as collecting sites for three reasons. First, these locations were known to have well established populations of *L. hesperus*. Second, these locations enabled comparisons between individuals collected from two very different host plants – strawberry and alfalfa – which would allow us to investigate genetic differentiation by host. Finally, geographic distance was also an important consideration in our experimental design and sampling to address the unanswered question of migration and gene flow among geographically isolated populations at both relatively near and far distances. Sample sizes in the final analyses (Table 1) are indicative of two things – the number of individuals collected at each location and the success of DNA extraction of individuals from each location. For example, individual *L. hesperus* were more rare in samples collected in Camarillo, CA as compared to samples collected in Watsonville, CA (Table 1).

**Table 1:** Collection information for populations of *Lygus hesperus* Knight in this study.

Locality	Collection Date	Host Plant	No. of Individuals
Othello, WA	August, 2009	Alfalfa	21
Prosser, WA	August, 2009	Alfalfa	16
Touchet, WA	August, 2009	Alfalfa	17
Parma, ID	August, 2009	Alfalfa	5
Parma, ID	July, 2009	Alfalfa	10
Santa Maria, CA	August, 2009	Strawberry	2
Prunedale, CA	August, 2009	Strawberry	19
Camarillo, CA	May, 2010	Strawberry	7
Camarillo, CA	May, 2010	Strawberry	2
Camarillo, CA	October, 2009	Strawberry	3
Camarillo, CA	November, 2009	Strawberry	1
Camarillo, CA	December, 2009	Strawberry	1
Camarillo, CA	June, 2010	Strawberry	7
Watsonville, CA	September, 2010	Strawberry	6
Watsonville, CA	September, 2010	Strawberry	2
Watsonville, CA	September, 2009	Strawberry	15

Individual insects were morphologically identified in the laboratory using a field key to common *Lygus* species in California [16]. As a first sort, we used only those bugs sampled that were considered to be *L. hesperus* using the field key in the DNA extraction and genetic sampling. All other Lygus species were thus potentially excluded from our genetic analyses. This was done as a first step to check the accuracy of our morphological identification methods in the field and laboratory against the genetic data we planned to collect. Far more western tarnished plant bugs were collected for our study than were used in the genetic anaylses. No specific permits were required for the described field collections. Locations where the insects were collected belonged to our agricultural cooperators as well as to either Washington State University or the Univ. of California-Davis. These locations are not protected in any way and the species collected were also not endangered or protected. Bugs were placed in 95% ethanol and stored at −20°C for DNA extraction, sequencing and analysis. Two sequences from GenBank (*L. lineolaris* AY252909 and *L. elisus* AY253038) were used as outgroups. According to the record in Genbank, these two sequences were directly submitted to Genbank by Wheeler and Schuh in 2003 but were unpublished at the time of submission. Five voucher specimens of *L. hesperus* from each location were deposited in the Washington State University M.T. James Entomological Collection.

### DNA Extraction

In order to develop and implement a standardized DNA region as a tag for rapid and accurate species identification for Lygus across the western USA, we amplified a 452 bp region of the mtDNA COI gene. We extracted genomic DNA using a variety of methods. The most cost-effective method was the Chelex®100 sodium form (BIO-RAD Chelex 100 Molecular Biology Grade Resin). However, the Promega Wizard® Genomic DNA purification Kit (Promega, Madison WI) and the QIAGEN DNeasy Blood & Tissue Kit (QIAGEN Inc. -USA, Valencia CA) were also used for some individuals, following the manufacturers' recommended protocols. The Chelex 100 protocol was modified and was conducted as follows. Each specimen was put whole into an empty 0.2 ml micro centrifuge tube, and ground in liquid nitrogen into powder. Initially, only legs were used but the quality of DNA was too poor for sequencing, therefore we used whole bodies. Then 200 ul of 20% Chelex 100 solution was added to the tube, followed by 2 ul of 20 mg/ml Proteinase K solution. The tube was then incubated at 95°C for 20 minutes, centrifuged at 16,000×g for 3 minutes and the supernatant collected for use in PCR. For specimens that were limited in number from a location and/or host plant, we used the Wizard or QIAGEN DNEasy methods following the manufacturer's protocols.

### DNA Analysis

To identify primers that would amplify the mtCOI-5P region from Lygus species, a tBlastx search on the NCBI website was performed using the *L. lineolaris* (AY252909) sequence as a query. The first 21 nucletoide sequences with signficant expect values were then used in a Clustal W alignment in MegAlign (Table S1; Lasergene, Madison, WI). The consensus sequence was used to design sequence specific primers with the online program Primer3 (http://biotools.umassmed.edu/bioapps/primer3_www.cgi), and purchased from Integrated DNA Technologies®. Ten primer pairs were assayed but consistent and strong amplification of the predicted size PCR product from our Lygus samples was produced with the forward primer (5′- CCA GGA TCA TTT ATT GGA GAT GA-3′) and reverse primer (5′- GAT AGG ATC TCC CCC TCC TG -3′) and these were used for subsequent PCR amplification of all samples. Sequence-specific primers such as these, despite having a mismatch in nucleotide sequence with one or more positions in several of the 21 species used in the alignment, nevertheless, were predicted to reliably sequence the mtCOI-5P region from individuals in the genus Lygus but also from members of other Mirid and Anthocorid species. This was important in our study, to amplify and thus detect divergent sequences. PCR was done in a 25 ul reaction volume containing 16.5 µl ddH_2_O, 2.5 ul 10× PCR buffer (Invitrogen, Life Technologies, Carlsbad CA), 1.25 µl 50 mM MgCl_2_ (Invitrogen, Life Technologies, Carlsbad CA), 0.5 µl 10 mM dNTPs (Invitrogen, Life Technologies, Carlsbad CA), 0.125 µl DMSO, 0.125 µl Taq (Invitrogen, Life Technologies, Carlsbad CA), 1 ul 20 ng/µl DNA, and 1.25 µl of 250 nM forward and reverse primers. Amplifications were completed in an ABI 100 Programmable Thermal Controller under the following cycling parameters: 95°C for 10 min, 35 cycles of 95°C for 30 s′, 55°C for 30 s, and 72°C for 60 s, with a final extension period of 10 min at 72°C, then set 4°C to hold.

PCR products were visualized after electrophoresis on 1% agarose gels stained with ethidium bromide, purified using Freeze ′N Squeeze DNA Gel Extraction Spin Columns (Bio-Rad, Hercules CA). Amplifications for each individual were directly sequenced using the same forward and reverse primers described above for PCR amplification. Each individual PCR product was thus sequenced in both directions using BigDye (ABI BigDye TM v.3.1 Cycle Sequencing) at the Washington State University Molecular Biology Core Laboratory. Sequences from both reads per individual were aligned to generate a consensus and a quality score was assigned to each position by using EditSeq (Lasergene, Madison WI) and Seqman (Lasergene, Madison WI). Sequences were trimmed to 447 bp. When necessary, manual editing of sequences was conducted by comparing electropherograms. Thirty-one new mtCOI haplotype sequences were deposited in GenBank and accession numbers are given in Table S2.

### Genetic Analysis

The consensus sequences for 134 individuals and 2 outgroup sequences were assembled. Sequences of the 2 outgroup species were obtained from GenBank: *L. lineolaris* (AY252909) and *L. elisus* (AY253038). To analyze the COI gene sequence and to identify species-specific sequences, the raw sequences for each sample were aligned using Clustal W in MegAlign (Lasergene, Madison WI).

The relationship between our presumed *L. hesperus* individuals and two other species, *L. lineolaris* and *L. elisus*, was investigated by reconstructing phylogenetic trees. Further Maximum Parsimony (MP), Neighbor Joining (NJ), Maximum Likelihood (ML), Minimum Evolution (ME), UPGMA, and posterior probability analyses of the data were performed in MEGA5 Beta6.1 [17] and MrBayes3.1.2 [18] to produce robust and statistically supported phylogenetic trees and phenetic clustering diagrams. The information was used as the basis for comparison of the unknown *Lygus* samples and to assess whether sequences from the proposed barcode regions form species-specific clusters.

Posterior probability analysis was carried out by using the parallel version of MrBayes3.1.2 [18] under the best-fit model. jModeltest0.1.1 [19], [20] was used to determine the best fit model for the MrBayes analysis. The best-fit substitution TPM2uf+I+G Bayesian Inference model was selected for posterior probability analysis. No gaps were necessary in the region of COI (447 bases) used. The TPM2uf+I+G model parameters of each process partition were set to be independent. Each search was run with the following settings: four chains (three heated using the default setting); random starting tree; sampling every 100 generations. Trees corresponding to the first 25% of generations were discarded as the burn-in and the remainder used to construct a 50% majority rule consensus tree. Starting trees for each chain were random and the specific values for the analysis of MrBayes3.1.2 were chosen by jModelTest0.1.1. Each metropolis coupled MCMC was run for 3,000,000 generations. By this time the chains had always converged to stable likelihood values <.0.01. Posterior probabilities were used to assess clade support. Log likelihood scores were plotted across generations to determine stationarity. The harmonic means of likelihoods were estimated using the sump command in MrBayes3.1.2, and these were used to compute Bayes factor comparisons across partitioning strategies and model assignments. Bayesian topology and branch posterior probabilities were computed by majority rule consensus after removing all “pre-burn-in” trees.

*L. hesperus* mitochondrial haplotypes and haplotype diversity were calculated using the program ARLEQUIN [21]. Haplotype networks were constructed using the statistical parsimony method of Templeton et al. [22] in the software programs TCS, version 1.21 [23] and Network 4.5.1.0 [24]. COI sequences of specimens (Table 1) were grouped according to collection location for a total of eight geographic locations. Genetic differences between these populations were approximated by calculating estimates of F_st_ for each pair of groups by using the program ARLEQUIN [21]. The significance of F_st_ values was evaluated by permuting the haplotypes (99,999 permutations) between groups. Sequential Bonferroni corrections were applied. Partitioning of genetic variation was estimated using the analysis of molecular variance (AMOVA) implemented in ARLEQUIN. Genetic variation was partitioned into two levels: 1) within populations of *L. hesperus* and 2) among populations of *L. hesperus*. The significance of population differentiation was evaluated using the permutation method (99,999 permutations) invoked in ARLEQUIN.

## Results

We genotyped 134 specimens representing *Lygus* bugs from three locations in Washington alfalfa, four locations in California strawberry, and one location from an Idaho alfalfa field. A 447 bp region of the mitochondrial COI gene was used for all analyses. Of 134 unique *Lygus* specimens, 77 individuals had identical sequences for this region comprising 57.5% of the total sample. In addition, several sequences were shared by more than one individual.

### Phylogenetic Relationships

**Figure 2:**
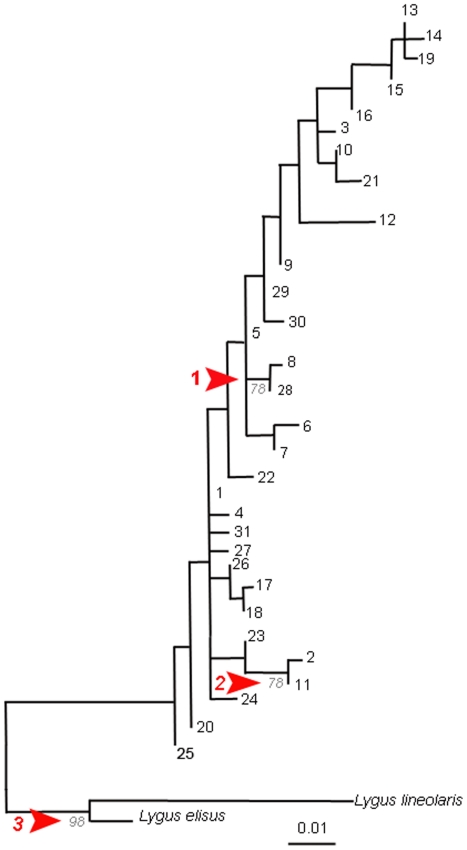
A ML tree representing a common topography of the 31 *L. hesperus* haplotypes identified in this study. Lineolaris refers to *L. lineolaris*, Elisus refers to *L. elisus*, and all numbers at the end of the branches represent haplotypes whereas numbers at the three branches indicate bootstrap support. The three arrows label strong bootstrap support at the nodes indicated. Sequence divergence is noted by the ruler.

Phylogenetic and clustering analyses were conducted using MP, ML, ME, NJ and UPGMA in MEGA5 BETA 6.1. Posterior probability analyses were conducted in MrBayes 3.1.2. Within the 447 bp region, there were 20 polymorphic sites, 19 of which were parsimony informative. All analyses were similar and a ML tree was produced using all 31 haplotypes (Figure 2). All haplotypes of presumed *L. hesperus* formed a monophyletic clade, distinct from the two outgroup species (Figure 2; Table 2).

**Table 2:** Support values from all phylogenetic analyses for the three well-supported branches of the phylogeny in Figure 1.

Clade	Bootstrap Value					Posterior Probability
	ME	MP	ML	NJ	UPGMA	Mr. Bayes
1	79	75	78	81	76	0.99
2	96	89	78	96	89	0.97
3	94	82	98	93	91	1.00

### Genetic Diversity

Thirty-one different mitochondrial haplotypes (Table S2) were found among the 134 specimens. Seventy-seven individuals shared the most common haplotype present in all eight populations sampled. Of the 31 haplotypes, 20 were unique and were represented by only one individual. In the other 11 haplotypes, eight were mixtures of individuals from different populations while only three were found to occur in single populations (Tables 3, 4).

**Table 3:** The total number of haplotypes found at each collection site.

	No. of Haplotypes
Othello, WA	7
Prosser, WA	6
Touchet, WA	6
Parma, ID	8
Santa Maria, CA	1
Prunedale, CA	3
Camarillo, CA	12
Watsonville, CA	7

**Table 4:** Geographic distribution of haplotypes with more than one individual.

Haplotype	1	2	4	5	6	8	11	13	20	21	22
Othello, WA	9	3			1		1				
Prosser, WA	13				3			1			
Touchet, WA	9				3			1			
Parma, ID	9		1				2	1	1		2
Santa Maria, CA	2										
Prunedale, CA	15			1		2					
Camarillo, CA	9			1		2			1		
Watsonville, CA	11		2	2		2			1	2	

### Gene Flow

Estimates of F_st_ between our geographically defined populations of *L. hesperus* ranged from −0.23686 to 0.13460 (Table 5). Among these F_st_ values, only three out of 28 pairwise population comparisons were significantly different from zero (Table 4). Significant pairwise F_st_ differences were found between the populations of Prunedale and two Washington populations (Tables 5, 6). The majority of the variation was found to be within populations with AMOVA (Table 6; 95.98%; df = 126, P<0.001). To analyze gene flow among different locations, we conducted further analyses in ARLEQUIN and obtained M values (Table 7). A value greater than 2 indicates gene flow between two populations [25]. All our M values were greater than 2, and nearly half of them were infinite. To analyze sequence divergence between populations, we conducted a pairwise comparison between different locations and with both outgroups using the Kimura 2 Parameter model [25]. Differences between populations were no greater than 1.2%, indicative of within species level divergence whereas the comparison of *L. hesperus* populations with *L. lineolaris* and *L. elisus* was from 4.5–4.7% indicative of species level sequence divergence (Tables 8–9) [11].

**Table 5:** Pairwise estimates of F_st_ between *L. hesperus* populations from California, Washington, and Idaho, *P<0.05, **P<0.01.

	Othello, WA	Prosser, WA	Touchet, WA	Parma, ID	Santa Maria, CA	Prunedale, CA	Camarillo, CA
Prosser, WA	0.00149						
Touchet, WA	0.01290	−0.02736					
Parma, ID	−0.03173	−0.00667	0.00135				
Santa Maria, CA	−0.14212	−0.16996	−0.11304	−0.19728			
Prunedale, CA	0.10075*	0.06285	0.13460*	0.05825	−0.21539		
Camarillo, CA	0.04568	0.00981	0.06690	0.01217	−0.19777	−0.02410	
Watsonville, CA	0.06353*	0.02550	0.07647	0.02630	−0.23686	−0.01261	−0.02448

**Table 6:** AMOVA table describing relationships among populations and within populations of *L. hesperus* Knight (p-values for va, vb and vc are: 0.02053, 0.67937, 0.09384. *P<0.05, **P<0.01).

Source of variation	d.f.	Sum of squares	Variance components	% of variation	Fixation Indices
Among states	2	10.855	0.10522 va	5.77	0.05772*
Among populations within groups	5	6.244	−0.03200 vb	−1.76	−0.01863
Within populations	126	220.483	1.74986 vc	95.98	0.04016
Total	133	237.582	1.82309		

**Table 7:** Matrix of M values (M = Nm for haploid data) describing predicted gene flow between populations of *L. hesperus*.

	Othello, WA	Prosser, WA	Touchet, WA	Parma, ID	Santa Maria, CA	Prunedale, CA	Camarillo, CA
Prosser, WA	334.49292						
Touchet, WA	38.24750	Infinite					
Parma, ID	Infinite	Infinite	369.41042				
Santa Maria, CA	Infinite	Infinite	Infinite	Infinite			
Prunedale, CA	4.46293	7.45571	3.21481	8.08394	Infinite		
Camarillo, CA	10.44452	50.46418	6.97356	40.57399	Infinite	Infinite	
Watsonville, CA	7.36977	19.10747	6.03813	18.51290	Infinite	Infinite	Infinite

**Table 8:** Pairwise distances of *L. hesperus* populations and *L. lineolaris/L. elisus* (outgroups).

	Othello, WA	Prosser, WA	Touchet, WA	Parma, ID	Santa Maria, CA	Prunedale, CA	Camarillo, CA	Watsonville, CA
Prosser, WA	1.0							
Touchet, WA	1.2	1.1						
Parma, ID	1.0	0.9	1.2					
Santa Maria, CA	0.7	0.6	0.9	0.6				
Prunedale, CA	0.8	0.6	1.0	0.7	0.2			
Camarillo, CA	0.9	0.8	1.0	0.8	0.3	0.4		
Watsonville, CA	0.9	0.8	1.0	0.8	0.3	0.4	0.6	
Outgroups	4.5	4.6	4.7	4.6	4.6	4.6	4.6	4.7

**Table 9:** Pairwise distances within populations of *L. hesperus*.

	% Difference
Othello, WA	1.06
Prosser, WA	0.92
Touchet, WA	1.36
Parma, ID	0.99
Santa Maria, CA	0
Prunedale, CA	0.3
Camarillo, CA	0.6
Watsonville, CA	0.56

## Discussion

The western tarnished plant bug is a major economic pest in the western United States. Despite tremendous variation in color pattern and diverse habitat associations among populations, this study revealed no significant differences in genetic structure among populations of the western tarnished plant bug. Population genetic analyses revealed the existence of 31 mitochondrial haplotypes in individuals from eight populations in California, Washington, and Idaho. COI sequence data for 134 specimens across these three states indicates significant levels of admixture and therefore insignificant levels of genetic structure by location and by host plant. Importantly, these data also support accurate identification of these morphologically variable bugs using widely available field keys [16]. These data support the idea that *L. hesperus* has undergone a relatively recent range and host plant expansion due to changes in agricultural environments over the last 100 years. Irrigated agriculture in the western USA drastically changed the landscape and host plant availability for polyphagous pests such as the western tarnished plant bug [26]–[28].

### Genetic structure

Our experimental specimens were collected from a relatively wide geographical range in the western United States – Washington, Idaho, and California. Within each state, locations were chosen so as to be relatively distant from each other (greater than 8 km), to increase the probability that we would detect genetic structure either by geographic location or by host plant. The *L. hesperus* bugs in our sample clustered together into a single clade (Figure 1). The most common haplotype was haplotype 1. Haplotype 1 consists of a mixture of 77 individuals from all locations found on both alfalfa and strawberry plants. This haplotype is shared by 57.5% of the entire sample. Indeed, the majority of haplotypes with greater than one individual consist of individuals from more than one population (Table 4). Each population also comprised multiple haplotypes with many haplotypes shared across location (Table 4), indicating that there were no population specific differences.

The distributions of the COI haplotypes suggest that gene flow between geographically isolated populations of *Lygus* species was not limited by location or host plant. Differences among haplotypes was found to be less than 2%, except for haplotype 13 (three individuals) and three singleton haplotypes – 14, 15, and 19 (Table 7). These four haplotypes were all found on alfalfa. In the phylogenetic analyses, the haplotypes clustered together, but were not statistically different from the other haplotypes (Figure 1). Most of the variation observed in the samples is within population (95.98%; Table 5). Only 5.77% of the variation was found among the three states. Therefore, at least for the mtCOI region we sampled, *L. hesperus* from Washington, Idaho and California are genetically similar enough to be considered as members of one species and have no significant genetic differentiation based on distance or host plant (Tables 8–9) [11], [25].

Within the *L. hesperus* clade, two branches were found to be more different from the other haplotypes. They were haplotypes 2, 8, 11 & 28 (Figure 1). Haplotype 2 is represented by three individuals from Othello, Washington collected from an alfalfa field. Haplotype 11 included one individual from Parma, Idaho, and one bug from Othello, Washington, both of which were collected from alfalfa plants. Haplotype 8 consisted of individuals collected in three different California strawberry fields and haplotype 28 was unique in representing a single individual from a Camarillo, California strawberry field. These rare haplotypes suggest that some genetic divergence may be occurring but from these limited data, no conclusions can be drawn for associations by location or host plant.

### Patterns of genetic divergence

Given that our phylogenetic analysis strongly supported a single *L. hesperus* lineage (Figure 1), we looked more closely at population level genetic structure for *L. hesperus*. Overwhelmingly the results suggest that these populations share haplotypes and are genetically mixing (Table 5). In addition, we found no significant patterns in the distribution of the 31 haplotypes by location or host plant when we compared all of the samples in our study. However, we did find evidence for limited gene flow between two Washington alfalfa fields and two California strawberry populations: (1) Othello, WA (alfalfa) and Prunedale, CA (strawberry), (2) Othello, WA (alfalfa) and Watsonville, CA (strawberry) and (3) Touchet, WA (alfalfa) and Prunedale, CA (strawberry; Table 5). The admixture suggested by our genotyping of the mtCOI barcode regions was surprising, as previous research [29] suggested that *L. hesperus* do not migrate more than a few miles from perennial crop fields where they are found in Washington state. In light of this data and the ontogenetic and geographical variation seen in *L. hesperus* populations, we suggest, based on historical patterns, a recent radiation of *L. hesperus* from a single, mixing source population. Based on the distribution of haplotypes found in our study, western tarnished plant bugs appear to be fairly good dispersers.

In summary, our results show that DNA barcodes can be very effective in identifying morphologically questionable or similar insects. But the importance of a solid taxonomic foundation for such applications of DNA barcoding cannot be overemphasized. We hope our research will help with the development of DNA barcode libraries in general and support future molecular research on *Lygus hesperus*. As a generic technology, barcoding can reduce the reliance on, and the need to be equipped for, a large number of taxon-specific methods [13]–[14], [30]. We also hope that the establishment of the DNA barcode information could help both taxonomists and non-taxonomists to accurate identify insects.

## Supporting Information

Table S1 Genbank accession numbers of the 21 mtCOI sequences used in the alignment for the Heteropteran: Cimicimorpha mtCOI primers designed in this study.
(DOCX)

Table S2 GenBank Accession numbers. A list of the individual GenBank Accession numbers for each haplotype.
(DOCX)
