# Supplementary material for: Identification of Lygus hesperus by DNA Barcoding Reveals Insignificant Levels of Genetic Structure among Distant and Habitat Diverse Populations
Source: PLoS One. 2012 Mar 30;7(3):e34528. doi: 10.1371/journal.pone.0034528 (PMC3316671; doi:10.1371/journal.pone.0034528)
Supplement: Table S1 — Genbank accession numbers of the 21 mtCOI sequences used in the alignment for the Heteropteran: Cimicimorpha mtCOI primers designed in this study. (DOCX) [file pone.0034528.s001.docx]

Supporting Information Table S1. Genbank accession numbers of the 21 mtCOI sequences used in the alignment for the Heteropteran: Cimicimorpha mtCOI primers designed in this study. Sequence order from top to bottom reflects the actual sequence alignment which was based on the results of the expect values from a tblastx search.

| Family | Species | Genbank Accession Number |
| --- | --- | --- |
| Miridae | *Lygus lineolaris* | EU401991 |
|  | *Lygus lineolaris* | AY252909 |
|  | *Lygus elisus* | AY253038 |
|  | *Stenotus binotatus* | AY252980 |
|  | *Knightomiris distinctus* | AY253052 |
|  | *Phytocoris* sp. | AY252990 |
|  | *Adelphocoris lineolatus* | AY252979 |
|  | *Capsus ater* | AY252977 |
|  | *Phytocoris ramosus* | AY253065 |
|  | *Oncerometopus* sp. | AY253036 |
|  | *Dichrooscytus* sp. | AY252986 |
|  | *Leptopterna dolobrata* | AY252983 |
|  | *Proba* sp. | AY253054 |
|  | *Phytocoris vanduzeei* | AY253064 |
|  | *Irbisia pacifica* | AY253082 |
|  | *Semium hirtum* | AY252921 |
|  | *Pycnocoris ursinus* | AY253099 |
|  | *Pycnocoris* sp. | AY253075 |
|  | *Neurocolpus arizonae* | AY253050 |
|  | *Oligotylus cercocarpicola* | AY253073 |
| Anthocoridae | *Orius niger* | EU427341 |
|  |  |  |
