# Supplementary material for: Identification of Lygus hesperus by DNA Barcoding Reveals Insignificant Levels of Genetic Structure among Distant and Habitat Diverse Populations
Source: PLoS One. 2012 Mar 30;7(3):e34528. doi: 10.1371/journal.pone.0034528 (PMC3316671; doi:10.1371/journal.pone.0034528)
Supplement: Table S2 — GenBank Accession numbers. A list of the individual GenBank Accession numbers for each haplotype. (DOCX) [file pone.0034528.s002.docx]

Supporting Information Table S2. Genbank accession numbers of the 31 *L. hesperus* mtCOI haplotypes identified in this study.

| Haplotype | Genbank Accession Number |
| --- | --- |
| 1 | JF823520 |
| 2 | JF823521 |
| 3 | JF823522 |
| 4 | JF823523 |
| 5 | JF823524 |
| 6 | JF823525 |
| 7 | JF823526 |
| 8 | JF823527 |
| 9 | JF823528 |
| 10 | JF823529 |
| 11 | JF823530 |
| 12 | JF823531 |
| 13 | JF823532 |
| 14 | JF823533 |
| 15 | JF823534 |
| 16 | JF823535 |
| 17 | JF823536 |
| 18 | JF823537 |
| 19 | JF823538 |
| 20 | JF823539 |
| 21 | JF823540 |
| 22 | JF823541 |
| 23 | JF823542 |
| 24 | JF823543 |
| 25 | JF823544 |
| 26 | JF823545 |
| 27 | JF823546 |
| 28 | JF823547 |
| 29 | JF823548 |
| 30 | JF823549 |
| 31 | JF823550 |
